# Identification of Novel Lung Cancer Driver Genes Connecting Different Omics Levels With a Heat Diffusion Algorithm

**DOI:** 10.3389/fcell.2022.825272

**Published:** 2022-01-26

**Authors:** Fei Yuan, Xiaoyu Cao, Yu-Hang Zhang, Lei Chen, Tao Huang, ZhanDong Li, Yu-Dong Cai

**Affiliations:** ^1^ Department of Science and Technology, Binzhou Medical University Hospital, Binzhou, China; ^2^ Department of Neurology, Binzhou Medical University Hospital, Binzhou, China; ^3^ Channing Division of Network Medicine, Brigham and Women’s Hospital, Harvard Medical School, Boston, MA, United States; ^4^ College of Information Engineering, Shanghai Maritime University, Shanghai, China; ^5^ Bio-Med Big Data Center, CAS Key Laboratory of Computational Biology, Shanghai Institute of Nutrition and Health, University of Chinese Academy of Sciences, Chinese Academy of Sciences, Shanghai, China; ^6^ CAS Key Laboratory of Tissue Microenvironment and Tumor, Shanghai Institute of Nutrition and Health, University of Chinese Academy of Sciences, Chinese Academy of Sciences, Shanghai, China; ^7^ College of Food Engineering, Jilin Engineering Normal University, Changchun, China; ^8^ School of Life Sciences, Shanghai University, Shanghai, China

**Keywords:** lung cancer, driver gene, epigenomics, genomics, transcriptomics, posttranscriptomics, heat diffusion algorithm, protein-protein interaction network

## Abstract

Cancer driver gene is a type of gene with abnormal alterations that initiate or promote tumorigenesis. Driver genes can be used to reveal the fundamental pathological mechanisms of tumorigenesis. These genes may have pathological changes at different omics levels. Thus, identifying cancer driver genes involving two or more omics levels is essential. In this study, a computational investigation was conducted on lung cancer driver genes. Four omics levels, namely, epigenomics, genomics, transcriptomics, and post-transcriptomics, were involved. From the driver genes at each level, the Laplacian heat diffusion algorithm was executed on a protein–protein interaction network for discovering latent driver genes at this level. A following screen procedure was performed to extract essential driver genes, which contained three tests: permutation, association, and function tests, which can exclude false-positive genes and screen essential ones. Finally, the intersection operation was performed to obtain novel driver genes involving two omic levels. The analyses on obtained genes indicated that they were associated with fundamental pathological mechanisms of lung cancer at two corresponding omics levels.

## Introduction

Driver gene is a commonly used description in oncology to describe genes with abnormal alterations that initiate or promote tumorigenesis (Pao and Girard, 2011; Tokheim et al., 2016). Identifying driver genes can help us reveal the fundamental pathological mechanisms of tumorigenesis (Pao and Girard, 2011). During tumorigenesis, genes may have pathological changes at different omics levels, including but not restricted to genomics (as DNA sequence alterations), epigenomics (as methylation status or other DNA modification status alterations), transcriptomics, and proteomics (Cancer Genome Atlas Research Network, 2014; Huang et al., 2015; Li et al., 2018). Using current biological techniques is time consuming but can detect all pathological alterations associated with tumorigenesis. However, not all alterations are associated with driver genes. Genes that are mutated or abnormally regulated during tumorigenesis but not associated with the initiation or progression of tumors are summarized as passenger genes (Pon and Marra, 2015). Most of the genes altered during tumorigenesis are actually passenger genes, which cannot help us understand or reveal the pathological mechanisms of cancer. Therefore, the identification of driver genes is one of the major research directions in oncology.

Based on traditional experiments, only hot spot genes, which have high mutation rate in sporadic cancer cases or have clear and obvious family hereditary histories, can be screened as candidates for cancer driver genes (Korenjak and Zavadil, 2019; Sears and Mazzone, 2020). For such pre-identified genes, driver potential needs to be validated using *in vitro* and *in vivo* experiments, which are quite time consuming and expensive (Chu et al., 2018; Sears and Mazzone, 2020), making it impossible and unreasonable for whole-omics wide screening. Therefore, to overcome the restrictions, scholars have introduced computational methods for discovering and pre-selecting candidate driver genes at whole omics level. Nowadays, identifying cancer driver genes using computational methods are not only effective but also reliable. At the beginning, computational methods are applied only for data at one omics level. With the development of algorithms and computational workflows, the integration of two or more omics levels of data to identify core cancer drivers has been realized (Turanli et al., 2018; Olivier et al., 2019). In 2016, Chen et al. (Chen et al., 2016) proposed shortest path-based method to identify novel lung cancer driver genes involving two omics levels. Later, Yuan and Lu employed another network algorithm, random walk with restart (RWR), to conduct the same investigation (Yuan and Lu, 2017). Several possible lung cancer driver genes involving two omics levels were proposed. This study continued the above two previous studies.

Cancer is one of the major threatening diseases for human health in the 21st century. As introduced above, identifying cancer biomarkers is one of the most effective way to explore the pathological mechanisms of tumorigenesis. However, revealing the potential biomarkers of all cancer subjects is difficult due to the limitation of data availability. In this study, we focused on one of the most common and deadly cancer subtype, namely, lung cancer. According to the statistics from GLOBOCAN estimates, in 2020, the general cumulative rates (0–75 years old) for lung cancer have been up to 3.78% in males and 1.77% in females, both of which are in the top of all cancer subtypes (rank 1 in males and rank 2 in females, following breast cancer) (Siegel et al., 2021). In 2021, more than 235,000 of new lung cancer cases and more than 130,000 of deaths from lung cancer are predicted in the United States alone (Siegel et al., 2021), confirming that lung cancer is a growing threat to humans in the 21st century.

Here, we integrated four levels of lung cancer omics data including epigenomics (methylation), genomics (gene variations), transcriptomics (gene expression), and post-transcriptomics regulation (microRNAs). Driver genes identified at each individual omics level were regarded as candidates for lung cancer drivers. Based on the candidates at each level, the Laplacian heat diffusion (LHD) (Carlin et al., 2017) algorithm was executed on a protein–protein interaction (PPI) network to identify raw driver genes. These genes were further filtered by a screen procedure, including permutation, association, and function tests, to exclude false-positive genes and select essential ones. Finally, intersection operation was carried out to identify driver genes involving at least two omics levels. Multiple genes were screened as potential multi-omics lung cancer drivers, and several genes were validated by recent publications via literature mining. The recognition of effective novel driver genes associated with lung cancer provided a novel approach for exploring the cancer pathological mechanisms of lung cancer and identifying clinical biomarkers.

## Materials and Methods

### Datasets

The driver genes at four omics levels, namely, epigenomics, genomics, transcriptomics, and post-transcriptomics regulation, were retrieved from a previous study (Chen et al., 2016). Genes were extracted from DNA methylation, somatic mutation, gene expression, and microRNA expression data, respectively, which were collected in TCGA (https://tcga-data.nci.nih.gov/docs/publications/luad_2014/) (Cancer Genome Atlas Research Network, 2014). The detailed data clean procedures can be found in Ref. (Chen et al., 2016). Finally, we obtained 153 driver genes at epigenomics level, 197 driver genes at genomics level, 1,373 driver genes at transcriptomics level, and 825 driver genes at post-transcriptomics level. Basing on these genes, we aimed to identify driver genes involving two levels.

### Network Construction

In this study, we used a powerful network algorithm, LHD algorithm (Carlin et al., 2017), to discover driver genes involving two omics levels. Thus, a network is necessary. PPI information is widely used to tackle protein- and gene-related problems (Ng et al., 2010; Chen et al., 2018; Zhang et al., 2019; Zhang and Chen, 2020; Liu et al., 2021; Pan et al., 2021a; Pan et al., 2021b; Zhu et al., 2021). Here, the human PPI information reported in STRING (Search Tool for the Retrieval of Interacting Genes/Proteins, http://www.string-db.org/, Version 10.0) (Szklarczyk et al., 2014) was adopted to construct a PPI network. To this end, we downloaded the file “9606. protein.links.v10. txt.gz”, containing 4,274,001 PPIs that involve 19,247 human proteins. Each PPI consists of two proteins, encoded by Ensembl IDs, and one confidence score with range between 1 and 999. Such score indicates the strength of the PPI. In fact, such score is obtained by considering several aspects of proteins, including close neighborhood in (prokaryotic) genomes, gene fusion, occurrence across species, gene coexpression, literature description, etc. As elaborated in the website of STRING, PPIs in STRING are derived from genomic context predictions, high-throughput lab experiments, (conserved) co-expression, automated textmining, previous knowledge in databases. Thus, they can widely measure the associations of proteins. This is the great advantage compared with the PPIs reported in other public databases. The constructed PPI network defined 19,247 proteins as nodes, and two nodes were connected by an edge if and only if they can constitute a PPI. Evidently, each edge in the network represented a PPI. To further indicate the difference of PPIs, we assigned each edge with a weight, which was defined as the confidence score of the corresponding PPI. For easy description, such PPI network was denoted by N.

### LHD-Based Method

Based on the PPI network N, an LHD-based method was designed to discover driver genes involving two omics levels. The method consisted of two stages. In the first stage, novel driver genes at each of four omics levels were identified by applying the LHD algorithm on the network N and performing a screen procedure. In the second stage, the novel driver genes at each level were refined to discover latent driver genes involving two omics levels.

#### LHD Algorithm

The LHD algorithm is a network diffusion algorithm (Carlin et al., 2017) that can deliver heat values on seed nodes to others in the network. Given a network *N*, let *A* be its adjacent matrix and *D* be the diagonal matrix, storing the degree of all vertices in the network. The Laplacian matrix *L* was defined as *D*-*A*, i.e., *L* = *D*-*A*. In addition, let *S* be the seed node set. The heat values on seed nodes are stored in a vector, denoted by $H\left(t_{0}\right)$ , and its length is equal to the number of nodes in the network *N*. Evidently, each component corresponds to one node, indicating the heat value of the node. In $H\left(t_{0}\right)$ , components corresponding to seed nodes are set to 1/|*S*|, and others are set to zero. With the pass of time, heat values on seed nodes are transmitted to other nodes in the following manner:

$$H\left(t\right)=H\left(t_{0}\right)\times e^{-Lt},$$ (1)

where *t* represents the time passed, and *H*(*t*) indicates the distribution of heat values at time *t*. Generally, as the time passes by, *H*(*t*) becomes stable. In reality, we tried several values of *t* and compared two consecutive vectors. If they were close enough, then the LHD algorithm was stopped. The final heat value distribution vector was selected as the outcome of the algorithm. Basing on this vector, we can extract the heat value of each node in the network. Nodes with high heat values were deemed to have strong associations with the seed nodes. By setting a proper threshold, important nodes can be selected.

For driver genes at one omics level, their encoding proteins were first obtained and fed into the LHD algorithm as seed nodes. The LHD algorithm was then performed on the PPI network N. According to the outcomes of the algorithm, nodes (proteins) assigned high heat values were selected as the raw driver genes at this level.

#### Screen Procedure

Some raw driver genes at each omics level can be obtained by executing the LHD algorithm. However, false-positive genes were inevitably included. A screen procedure was designed to control these genes.

##### Permutation Test

Heat value is very important to determine the selection of nodes (proteins). However, this value on some nodes (protein) was highly related to the structure of network *N*. Some nodes (proteins) more easily received high heat values, regardless of which nodes were seed nodes. Thus, the significance of heat value on each raw driver gene selected by the LHD algorithm should be further measured. For raw driver genes at each level, a permutation test was performed. In detail, we randomly generated 500 gene sets, which contained the same number of driver genes at this level. For each generated gene set, the LHD algorithm was executed on *N* with genes in this set as seed nodes. Finally, each raw driver gene was assigned a heat value. After all the randomly produced sets were considered, each raw driver gene was assigned 500 heat values. These values can be used to measure the significance of actual heat value that was obtained by driver genes at this level. A *p*-value was computed for each raw driver gene *g* as follows:

$$p-value\left(g\right)=\frac{Heat_{>}}{500},$$ (2)

where *Heat*_>_ denotes the number of randomly produced gene sets, on which the heat value of *g* is larger than its actual heat value. If a novel driver gene had a high *p*-value, then the heat values on several randomly generated gene sets were higher than its actual heat value, indicating that this heat value had no statistical significance. As such, this gene was not special for the driver genes at this level. Thus, we should select novel driver genes with low *p*-values. Given that 0.05 is always used as the cutoff to measure statistical significance, this study adopted it to filter novel driver genes at each level.

##### Association Test

By the permutation test, some false positive genes produced by the PPI network were excluded. To further select essential genes among remaining genes, we designed an association test. Several studies have reported that interacting proteins are more likely to share common functions. If the strength of the interaction was considered, then two proteins in a strong interaction were more likely to share common functions than those in a weak interaction. As mentioned in *Network construction*, the confidence score in STRING can measure the strength of an interaction and thus can be used to design the association test. For formulation, the confidence score on the interaction of proteins *p*_1_ and *p*_2_ was denoted by $S\left(p_{1},p_{2}\right)$ . A measurement, namely maximum association score (MAS), was calculated for each driver gene *g* by

$$MAS\left(g\right)=\max\left\{S\left(g,g^{\prime }\right)|g^{\prime }\text{ is a driver gene}\right\}$$ (3)

Clearly, the gene with a high MAS was more important and should be kept. We can set a high threshold of MAS to select essential driver genes.

##### Function Test

To further extract essential driver genes at each level, we designed a third test, namely, function test. This test was based on two types of functional terms: 1) gene ontology and 2) KEGG pathway. In general, driver genes at some level may be annotated by some common functional terms. If the latent one exhibited similar functional terms to some driver genes, then it had a high probability to be a novel driver gene. We first used enrichment theory (Subramanian et al., 2005) to measure the relationship between one gene and all functional terms. In detail, for one gene *g* and one functional term *f*, let *G* be the set consisting of *g* and its interacting genes and *F* be the set containing genes annotated by *f*. The enrichment score between *g* and *f* was defined as the -log_10_ of the hypergeometric test *p* value of *G* and *F*, which can be computed by

$$\text{Enrichment score}\left(g\text{,}f\right)=-\log_{10}\left(\sum_{k=m}^{n}\frac{\left(\begin{array}{c} M\\ k \end{array}\right)\;\left(\begin{array}{c} N-M\\ n-k \end{array}\right)}{\left(\begin{array}{c} N\\ n \end{array}\right)}\right)$$ (4)

where *N* stands for total number of human genes, *M* and *n* represent the number of genes in *F* and *G*, respectively, *m* denotes the number of common genes in *F* and *G*. The obtained values were collected in a vector, denoted by ES(g) for gene *g*. The linkage between two genes was measured according to their vectors by using the following equation:

$$\Psi \left(g_{1},g_{2}\right)=\frac{ES\left(g_{1}\right)\cdot ES\left(g_{2}\right)}{\left\|ES\left(g_{1}\right)\right\|\cdot \left\|ES\left(g_{2}\right)\right\|},$$ (5)

where *g*_1_ and *g*_2_ represent two genes, $ES\left(g_{1}\right)\cdot ES\left(g_{2}\right)$ denote the dot product of two vectors, and $\left\|ES\left(g_{1}\right)\right\|$ indicates the module of the vector. Evidently, higher values indicated stronger linkage of two genes. For each latent driver gene *g*, the maximum function score (MFS) was computed as follows:

$$MFS\left(g\right)=\max\left\{\Psi \left(g,g^{\prime }\right)|g^{\prime }\text{ is a driver gene}\right\}$$ (6)

Similar to MAS, genes with high MFS values were more likely to be novel driver genes. By setting a proper threshold, essential genes can be obtained.

#### Intersection Operation

Based on the LHD algorithm and a screen procedure, some essential novel driver genes were obtained at each omics level. By taking the intersection operation, we identified some driver genes involving two levels. As four levels were considered in this study, we finally obtained six driver gene sets. Genes in each set were deemed to be driver genes involving two omics levels.

## Results

**FIGURE 1:**
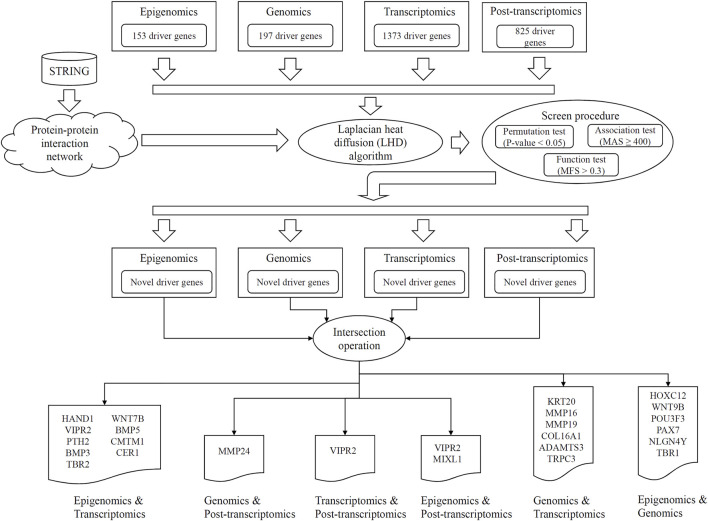
Entire procedures of LHD-based method for identification of multi-omics lung cancer driver genes. Based on driver genes at one omics level, the Laplacian heat diffusion (LHD) algorithm is executed on a protein–protein interaction network reported in STRING to identify raw driver genes. These genes are filtered by a screen procedure. The intersection operation is conducted to identify driver genes involving any two omics levels. Six groups are accessed, each of which contains driver genes involving two omics levels.

In this study, an LHD-based method was proposed to identify novel driver genes involving two omics levels. The entire procedures are illustrated in Figure 1. The detailed results at each procedure are presented in this section.

### Latent Driver Genes at Each Level

In the first stage of LHD-based method, some latent driver genes were identified for each omics level. For epigenomics level, 153 validated driver genes were fed into the LHD algorithm, which was executed on the PPI network *N*. Each node was assigned a heat value. We selected the nodes with heat value no less than 10^–5^ and obtained 13,101 nodes. The heat values of the selected nodes are provided in Supplementary Table S1. A screen procedure was then performed to filter essential candidates. First, a permutation test was adopted to determine the statistical significance of heat value on each selected node, resulting in a *p*-value for each node (Supplementary Table S1). A total of 311 nodes with *p*-value less than 0.05 were selected. Second, an association test was executed to test the importance of the 311 remaining nodes, assigning an MAS to each node (Supplementary Table S1). The threshold of MAS was set as 400, resulting in 228 nodes. Finally, function test was used to evaluate each remaining node, which was assigned with an MFS (Supplementary Table S1). The threshold of MFS was set as 0.3. A total of 199 nodes were obtained, and their corresponding genes were selected as the latent driver genes at epigenomics level.

For the three other levels, the same procedures with common thresholds for all measurements were performed. All measurements on each node at the three levels are provided in Supplementary Tables S2–S4. The numbers of remaining nodes at each stage of LHD-based method are listed in Table 1. As a result, 84, 174, and 39 latent driver genes were accessed at levels of genomics, transcriptomics, and post-transcriptomics regulation, respectively.

**TABLE 1:** Remaining latent driver genes at each stage of LHD-based method.

Level	LHD-algorithm	Permutation test	Association test	Function test
Epigenomics	13,101	311	228	199
Genomics	14,321	226	114	84
Transcriptomics	16,256	224	184	174
Post-transcriptomics	17,007	59	44	39

**FIGURE 2:**
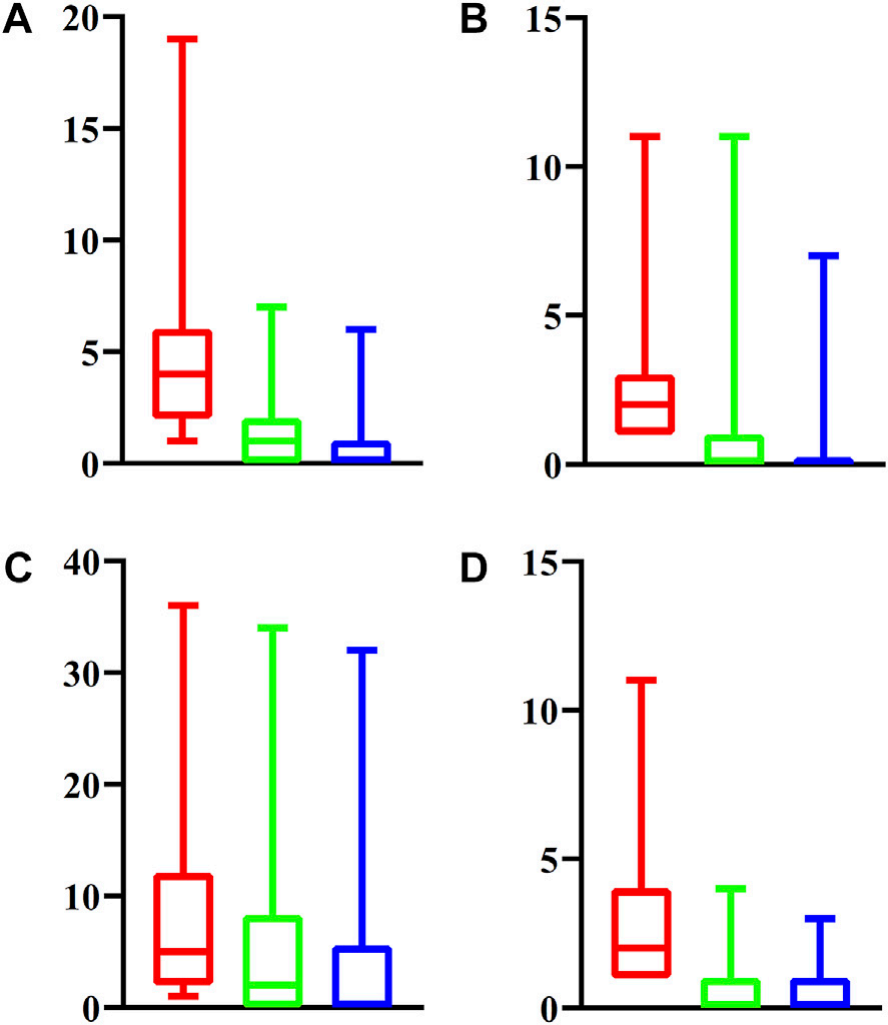
Boxplot to show the associations between latent driver genes and validated ones at each of four omic levels. **(A)** Epigenomics; **(B)** Genomics; **(C)** Transcriptomics; **(D)** Post-transcriptomics. The *Y*-axis represents the number of validated genes that can interact with latent genes with different strength. The red (green, blue, respectively) box denotes number of validated genes that can interact with latent genes with medium (high, highest, respectively) confidence.

For latent driver genes at each omics level, we further investigated their associations with validated genes mentioned in *Datasets*. For each omics level, the PPIs between latent and validated genes were extracted. For each latent gene, we counted three values, which were defined as the number of validated genes that can interact with the latent gene with medium confidence (≥400), high confidence (≥700) and highest confidence (≥900). These values of all latent genes at four omics levels were indicated by four boxplots, as shown in Figure 2. It can be observed that each latent gene can interact with at least one validated gene with medium confidence and several latent genes can interact with validated genes with high or highest confidence. This fact indicated that the relationships between latent and validated genes were quite close, increasing the probabilities of latent genes to be actual driver genes.

### Driver Genes Involving Two Levels

According to LHD-based method, we conducted the intersection operation of latent driver genes at two levels. Some driver genes involving two levels were obtained. The number of driver genes involving two levels is listed in Table 2, and the detailed genes for any two levels are also listed in Table 2. At least one driver gene was accessed for any two levels. *Discussion* presents an extensive discussion on some of the obtained genes.

**TABLE 2:** Driver genes involving two omics levels^a^.

Level	Epigenomics	Genomics	Transcriptomics	Post-transcriptomics
Epigenomics	-	6	9	2
Genomics	*HOXC12*, *WNT9B*, *POU3F3*, *PAX7*, *NLGN4Y*, *TBR1*	-	6	1
Transcriptomics	*HAND1*, *VIPR2*, *PTH2*, *BMP3*, *TBR2*, *WNT7B*, *BMP5*, *CMTM1*, *CER1*	*KRT20*, *MMP16*, *MMP19*, *COL16A1*, *ADAMTS3*, *TRPC3*	-	1
Post-transcriptomics	*VIPR2*, *MIXL1*	*MMP24*	*VIPR2*	-

a Numbers in the upper triangle part of this table represent the numbers of driver genes involving two omics levels.

### Comparison With Previous Results

Two previous studies (Chen et al., 2016; Yuan and Lu, 2017) reported some novel driver genes involving two omics levels. The comparison of driver genes indicated that the driver genes reported in the present study were completely different from those in previous two studies. The previous two studies adopted the shortest path-based and RWR-based methods, respectively, to discover novel driver genes, which had quite different principles and procedures; as such, the difference in the driver genes reported between the present study and previous studies was considered reasonable. On the other hand, each method has its limitations, and some driver genes may be omitted. The driver genes reported in this study can be essential supplements for the previous studies.

## Discussion

Novel driver genes involving two of the four omics levels (epigenomics, genomics, transcriptomics, post-transcriptomics levels) were identified using LHD-based method. Six groups of latent multi-omics driver genes for lung cancer were screened. According to recent publications, several identified multi-omics driver genes involving two of the four omics levels can be confirmed to be associated with fundamental lung cancer tumorigenesis-associated pathological mechanisms. The detailed discussion on the genes identified in each group can be seen below.

### Shared Genes Between Epigenomics and Post-transcriptomics Regulation

Two genes were identified to be lung cancer drivers at epigenomics (methylation) and post-transcriptomics regulation (microRNA) levels. The first gene is *VIPR2* (ENSP00000262178). Early in 2017, researchers from Sungkyunkwan University confirmed that *VIPR2* is associated with lung cancer at the DNA methylation level (Um et al., 2017). As for the microRNA level, no direct reports confirmed the correlation between lung cancer and *VIPR2*. However, such gene has been shown to be associated with pancreatic cancer tumorigenesis at the microRNA level (Naderi et al., 2014), indicating the specific role of the gene during tumorigenesis. Therefore, this gene could have regulatory effects on lung cancer at the microRNA level. For the next gene involving two omics levels, *MIXL1* (ENSP00000355775), an epigenome analyses in 2019 on circulating tumor cells associated with metastasis revealed that circulating tumor cells in lung cancer has typical epigenomic alterations in *MIXL1* (Gkountela et al., 2019). As for its regulatory effects at post-transcriptomics microRNA level, effective microRNAs from famous microRNA family, let-7 family, has shown to be associated with the dedifferentiation transformation of lung cells during embryonic development or malignant transformation (Navarro and Monzo, 2010). The gene *MIXL1* is regulated by microRNAs from let-7 family during the initiation and proliferation of lung cancer stem cells (Navarro and Monzo, 2010). Therefore, such gene is a potential biomarker presenting abnormal microRNA level regulation during lung tumorigenesis.

### Shared Genes Between Epigenomics and Genomics

Six genes were shown to be essential lung cancer drivers involving methylation and genomics levels. The first gene *HOXC12* (ENSP00000243103) has been reported to be triggered by its pathological methylated and inactivated promoter during lung tumorigenesis, validating its specific driver role at the methylation level (Guerrero-Preston et al., 2014). At the genomics level, researchers from Iran in 2019 reported that a SNP in gene *HOXC12* is associated with risk of multiple cancer subtypes, implying the specific lung cancer driver potentials of this gene (Hajjari and Rahnama, 2019). Genes such as *WNT9B* (ENSP00000290015) (Xu et al., 2019), *POU3F3* (ENSP00000355001) (Zeng et al., 2020), and *PAX7* (ENSP00000364524) (Rácz et al., 2000) are lung cancer biomarkers at different omics levels. As for their respective contribution at epigenomics and genomics levels, *WNT9B* (Lan et al., 2006; Farkas et al., 2014), *POU3F3* (Li et al., 2014; Kumar et al., 2016), and *PAX7* (Starzyńska et al., 2020) have all been shown to be associated with lung cancer at epigenomics and genomics levels independently. For the two remaining genes, *NLGN4Y* (ENSP00000342535) and *TBR1* (ENSP00000374205), in 2019, researchers from University of Southampton summarized *NLGN4Y* as a multi-omics level driver for lung cancer at least at epigenomics and transcriptomics levels (Jeyananthan and Niranjan, 2019). As for the genomics level, variants in *NLGN4Y* can regulate cell proliferation in multiple pathogenesis, though not directly reported in lung cancer (Nardello et al., 2021). The potential regulatory effects of such gene on cell proliferation indicated that it may also be associated with lung cancer at the genomic level. Similar experimental works support the driver role of the *TBR1* gene at genomics and epigenomics levels (Ischenko et al., 2014; Serth et al., 2020). Therefore, all predicted genes at epigenomic (methylation) and genomics levels have been validated to be potential lung cancer drivers.

### Shared Genes Between Epigenomics and Transcriptomics

Nine genes have been shown to be associated with lung cancer and are potential lung cancer drivers at epigenomics and transcriptomics levels. Among the nine genes, *VIPR2* (ENSP00000262178) has already been discussed above and shown to be regulated at epigenomics and post-transcriptomics level. According to the same supporting paper mentioned above, this gene can be regulated at the transcriptomics level (Um et al., 2017). Other genes, such as *TBR2* (ENSP00000295743) and *WNT7B* (ENSP00000341032), which are the homologues of *TBR1* and *WNT9B*, respectively, are also reasonable to be speculated as candidate regulators at epigenomics and transcriptomics levels. Considering the limitation of the manuscript’s length, we selected three candidates for detailed discussion: *HAND1*, *BMP3*, and *CMTM1*. According to recent publications, *HAND1* is a typical DNA methylation biomarker of small-cell lung cancers (Kalari et al., 2013). At the transcriptomics level, *HAND1* has been identified as a biomarker in a newly reported single-cell study (Yin et al., 2019). Apart from *HAND1*, *BMP3* together with *BMP5* has been considered potential multi-omics level lung cancer biomarkers according to recent publications. In 2015, researchers from Huazhong University of Science and Technology conducted transcriptomics level analyses on human lung squamous cell carcinoma, confirming the driver role of *BMP3* and *BMP5* at the transcriptomics levels (Deng et al., 2015). At the epigenomics level (methylation), an earlier study on colorectal cancer confirmed that the methylation alteration on *BMP3* or *BMP5* may trigger the malignant transformation of normal cells (Loh et al., 2008). Therefore, the two genes could be potential lung cancer biomarkers at multi-omics levels. The *CMTM1* gene has also been reported by two independent studies to be associated with lung cancer at transcriptomics (Hou et al., 2020) and methylation (Shao et al., 2007) levels, respectively. Other genes, such as *PTH2* (Kim et al., 1998), *TBR2* (Kaowinn et al., 2017), *WNT7B* (Kirikoshi and Katoh, 2002), and *CER1* (Semenova et al., 2016), either have similar effects shared with their homologues or have been independently reported to be associated with lung cancer at different omics levels.

### Shared Genes Between Genomics and Post-transcriptomics Regulation

Only one gene has been predicted to regulate lung cancer-associated pathological effects at genomics and post-transcriptomics levels. According to recent publications, *MMP24* (ENSP00000246186) is associated with lung cancer at different omics levels (Fontenele et al., 2015; Wang et al., 2019; Wang et al., 2020). At genomics and post-transcriptomics levels, variants on *MMP24* have been shown to be associated with lung cancer via a GWAS study in 2015 (Fontenele et al., 2015). In 2019, researchers from Tumor Hospital of Wuwei confirmed that microRNA-133a regulates *MMP24* and further contributes to the tumorigenesis of lung cancer (Wang et al., 2019).

### Shared Genes Between Transcriptomics and Post-transcriptomics Regulation

For genes that have been identified to be potential lung cancer drivers at transcriptomics and post-transcriptomics levels, only one gene, namely, *VIPR2* (ENSP00000262178), was identified as multi-omics level regulator. As discussed above, this gene has shown to be effective at epigenomics, transcriptomics, and post-transcriptomics. Therefore, this gene could be a multi-omics regulator.

### Shared Genes Between Genomics and Transcriptomics

Six genes have been identified to regulate lung tumorigenesis via genomics and transcriptomics levels. The first gene *KRT20* (ENSP00000167588) has been shown to define the invasive characteristics of cancer cells at the transcriptomics level in multiple cancer subtypes (Eckstein et al., 2018), including lung cancer (Mollaoglu et al., 2018; Maly et al., 2019). As for the genomics level, variants in *KRT20* are associated with lung cancer (Huang et al., 2015). Genes *MMP16* (ENSP00000286614) and *MMP19* (ENSP00000313437) belong to the matrix metalloproteinase family that is associated with multiple cancer subtypes, including lung cancer (Rudolph-Owen et al., 1998). As for their effects on genomics and transcriptomics levels, in the same publication, a summary of the effects of matrix metalloproteinases at multi-omics have been presented and demonstrated. *TRPC3* (ENSP00000368966), as another novel driver gene associated with lung cancer at genomics and transcriptomics levels, has been validated to be potential genomic and transcriptomic driver for lung cancer. In 2016, a researcher from Guangzhou Medical University validated the effects of *TRPC3* variants on lung cancer risk (Zhang et al., 2016). In 2021, such gene has been reported to be associated with malignant transformation at the transcriptomics level (Lin et al., 2021). The two remaining genes are *COL16A1* (ENSP00000362776) and *ADAMTS3* (ENSP00000286657). In 2016, *COL16A1* has been shown to be associated with lung tumorigenesis via specific mutant patterns validated by *in vitro* A549 cell lines (Wang et al., 2016). As for the transcriptomics level, researchers from Medical University of South Carolina validated that at least in oral squamous cell carcinoma, the alteration of the gene expression of *COL16A1* may promote tumor growth via interacting with the RNA-binding protein CELF1 (House et al., 2015). As for the last predicted gene *ADAMTS3*, according to recent publications, this gene has been confirmed to participate in lung tumorigenesis at genomics and transcriptomics levels (Vanni et al., 2016; Hannen et al., 2019).

Overall, several identified multi-omics lung cancer drivers were validated to be associated with fundamental pathological mechanisms. Therefore, LHD-based method was effective and accurate to identify lung cancer-associated tumor drivers and may help reveal the potential mechanisms of lung tumorigenesis.

### Clinical Applications on New Driver Genes

Based on recent publications, some identified multi-omics lung cancer drivers have been already applied for clinical use on the diagnosis or treatment against lung cancer. According to recent publications, *WNT9B*, as the multi-omics biomarker for lung cancer at both epigenomics and genomics levels has been shown to act as an effective drug target (Stewart et al., 2014). The application of such drug can significantly improve the survival probability for lung cancer (Stewart et al., 2014). The next widely reported gene is *MMP19*, as a new driver gene at both genomics and transcriptomics levels. It has been widely reported to be associated with tumorigenesis, including gastric cancer (Shen et al., 2020), glioma (Luo et al., 2018) and lung cancer (Yu et al., 2014). As for its clinical application for lung cancer diagnosis and treatment, *MMP19* has been reported to promote the metastasis of lung cancer and is associated with increased mortality of lung cancer as an active biomarker (Yu et al., 2014). *TRPC3*, as a driver gene at both genomics and transcriptomics levels have also been recognized as a promising clinical biomarker for lung cancer (Lastraioli et al., 2015), indicating the clinical significance of such multi-omics biomarker.

## Conclusion

This study investigated lung cancer driver genes involving two omics levels. An LHD-based method was proposed to identify multi-omics lung cancer driver genes. Several genes, such as *HOXC12*, *HAND1*, *VIPR2*, *KRT20*, *MMP24*, and *VIPR2*, were discovered, and their special roles at different omics levels of lung cancer were confirmed. These findings may help improve the research progress on lung cancer.

## Data Availability

Publicly available datasets were analyzed in this study. This data can be found here: https://tcga-data.nci.nih.gov/docs/publications/luad_2014/.
